# Racial/Ethnic Disparities in Exposure, Disease Susceptibility, and Clinical Outcomes during COVID-19 Pandemic in National Cohort of Adults, United States

**DOI:** 10.3201/eid2811.220072

**Published:** 2022-11

**Authors:** McKaylee M. Robertson, Meghana G. Shamsunder, Ellen Brazier, Mekhala Mantravadi, Rebecca Zimba, Madhura S. Rane, Drew A. Westmoreland, Angela M. Parcesepe, Andrew R. Maroko, Sarah G. Kulkarni, Christian Grov, Denis Nash

**Affiliations:** Institute for Implementation Science in Population Health, New York, New York, USA (M.M. Robertson, M.G. Shamsunder, E. Brazier, M. Mantravadi, R. Zimba, M.S. Rane, D.A. Westmoreland, A.M. Parcesepe, A.R. Maroko, S.G. Kulkarni, C. Grov, D. Nash);; Graduate School of Public Health and Health Policy, New York (M.G. Shamsunder, R. Zimba, A.R. Maroko^,^, C. Grov, D. Nash);; University of North Carolina at Chapel Hill Gillings School of Public Health and Carolina Population Center, Chapel Hill, North Carolina, USA (A.M. Parcesepe)

**Keywords:** COVID-19, coronavirus disease, severe acute respiratory syndrome coronavirus 2, SARS-CoV-2, coronaviruses, viruses, respiratory infections, racial/ethnic disparities, potential virus exposure, susceptibility, disease, healthcare access, healthcare disparities, clinical outcomes, serologic testing, social determinants of health, zoonoses, United States

## Abstract

We examined racial/ethnic disparities for COVID-19 seroconversion and hospitalization within a prospective cohort (n = 6,740) in the United States enrolled in March 2020 and followed-up through October 2021. Potential SARS-CoV-2 exposure, susceptibility to COVID-19 complications, and access to healthcare varied by race/ethnicity. Hispanic and Black non-Hispanic participants had more exposure risk and difficulty with healthcare access than white participants. Participants with more exposure had greater odds of seroconversion. Participants with more susceptibility and more barriers to healthcare had greater odds of hospitalization. Race/ethnicity positively modified the association between susceptibility and hospitalization. Findings might help to explain the disproportionate burden of SARS-CoV-2 infections and complications among Hispanic/Latino/a and Black non-Hispanic persons. Primary and secondary prevention efforts should address disparities in exposure, vaccination, and treatment for COVID-19.

Researchers have identified underlying medical conditions, comorbidities, older age, and male sex as biologic vulnerabilities for more severe COVID-19 outcomes (*1*,*2*). Evidence also suggests a disproportionate burden of COVID-19 infection, hospitalization, and death among Hispanic/Latino/a, Black non-Hispanic, and American Indian and Alaskan Native populations in the United States. (*3**–**6*). Early in the pandemic (March 2020), the Centers for Disease Control and Prevention (CDC) reported that twice as many Black persons were hospitalized because of COVID-19 than are proportionally represented in the United States. (*3*). Long-standing health and social inequities probably contribute to disparities in COVID-19 illness and death (*7**–**9*).

Public health interventions and policies with the potential to improve health might inadvertently amplify existing health disparities (*7*). Prevention efforts, such as social distancing or work from home policies, might have inequitable benefits across racial and ethnic groups because of differential employment in essential work settings or likelihood of living in multigenerational households (*7*,*8*,*10*). Less access to or use of healthcare also result in differential COVID-19 outcomes among racial and ethnic minority groups because later care presentation might limit treatment options (*6*,*8*). Blumenshine et al. proposed a pandemic disease model in which differences in exposure to the pathogen, susceptibility to severe illness if infected, and poor/delayed access to treatment might lead to disproportionate infection, illness, and death during a pandemic (*11*). To avoid exacerbating existing disparities, effective public health interventions and pandemic guidelines need to anticipate and mitigate the contribution of social determinants to disparities in exposure, susceptibility if exposed and access to treatment (*9*,*11*,*12*).

Our objective was to examine the influence of racial and ethnic differences in social determinants on COVID-19 outcomes within a large US national cohort of adults that was enrolled during the spring of 2020, the early phase of the COVID-19 pandemic. Using the Blumenshine model as a framework, we created 3 indices to assess social determinants: the ability to social distance as a measure of potential SARS-CoV-2 exposure, susceptibility to COVID-19 complications, and access to healthcare. We examined the relationship between each index with COVID-19 outcomes (COVID-19 hospitalization or seroconversion). Considering race/ethnicity as a social, rather than biologic construct (*13*), we assessed it as a potential effect measure modifier (EMM) of the relationship between each index and COVID-19 outcome.

## Methods

### Data Source and Population

The Communities, Households, and SARS-CoV-2 Epidemiology COVID Cohort Study is a geographically and sociodemographically diverse sample of adults (>18 years of age) residing in the United States or US territories who enrolled into a prospective cohort study during emergence of the COVID-19 pandemic in the United States (*14*). We used internet-based strategies to recruit a fully online cohort. We recruited study participants during March 28, 2020‒April 20, 2020, by advertisements on various social media platforms (e.g., Facebook) or by referral (anyone with knowledge of the study was allowed to invite others to participate). Internet-based strategies are effective for recruiting and following large and geographically diverse online cohorts and collecting at-home biological specimens (*15**–**17*). Details of cohort recruitment and follow-up been described by Robertson et al. (*14*). The study protocol was approved by the Institutional Review Board at the City University of New York (CUNY) Graduate School for Public Health and Health Policy.

### Variable Definitions

#### Race/Ethnicity

Respondents were asked: “Are you Hispanic, Latino/a, or Spanish origin?” and “Which of these groups would you say best represents your race?” Participants were then categorized as Hispanic/Latino/a, Black non-Hispanic, Asian/Pacific Islander non-Hispanic, White non-Hispanic, or other (which included participants identifying >1 race, along with those identifying as American Indian or Alaskan Native and other) (*18*). To reduce the number of participants in the other category, we used a hierarchical approach to assign participants to 1 of the predominant race/ethnicity groups in the United States, first categorizing all Black non-Hispanic and all multiracial participants who identified as Black (n = 103), and then categorizing the remaining multiracial participants as Asian/Pacific Islander non-Hispanic (n = 80) or White non-Hispanic (n = 1). The remaining 222 participants in the other category were participants who did not identify as Black, Asian, or White.

### Potential SARS-CoV-2 Exposure, COVID-19 Susceptibility, and Healthcare Access

We created 3 summative indices as proxies for potential SARS-CoV-2 exposure, susceptibility to COVID-19 complications, and difficulty with access to healthcare (*9*). We drew the indices and assessment items from a national survey that explored the experience of adults during the 2009–2010 influenza A(H1N1) pandemic (*9*,*19*). Specifically, the survey assessed disparities in H1N1 virus exposure, susceptibility to influenza complications, and access to healthcare during this influenza pandemic. We used the same exposure and access to care indices as the H1N1 survey and modified the susceptibility index to align with the conditions or exposures that CDC had identified in March 2020 as increasing the risk for COVID-19 complications. Each index was a summative score, in which a higher risk response was given a value of 1, and a lower or no risk response was given a value of 0. Therefore, a higher value would indicate a greater exposure risk, greater susceptibility, and greater difficulty with access to care and treatment.

First, as the measure of potential SARS-CoV-2 exposure, we included built-environment and work-related items that contributed to the ability to social distance. The built-environment items included living in an urban area, living in a multiunit dwelling (e.g., apartment building), and the ability to avoid public transportation. The work-related items included essential worker status and whether respondents were able to stay home from work or work from home, if needed. Specifically, respondents were asked to indicate yes, no or not applicable to the following statements: I am able to work at home; if I do not go to work because I am ill, I will not get paid for the time I am at home; I have sick leave at my job if I need to use it; I could lose my job or business if I am not able to go into work; my job can only be done in my workplace. Respondents who did not work were considered not at risk for the work-related items (i.e., a score of 0). Essential worker status was defined as having been involved in healthcare or other essential work (e.g., first responders) in the 2 weeks before the survey (*14*).

Second, as the measure of COVID-19 susceptibility, we used conditions or exposures that CDC had identified as increasing the risk for COVID-19 complications given SARS-CoV-2 infection in March 2020: age >60 years, daily smoking, and underlying chronic conditions (chronic lung disease including chronic obstructive pulmonary disease, emphysema, and chronic bronchitis; serious heart conditions including angina/coronary heart disease, high blood pressure, history of myocardial infarction; current asthma; type 2 diabetes; kidney disease; immunocompromised condition; or HIV positive). Finally, as the measure of healthcare access, we used factors that affect medical care access: no primary care doctor, concerns about the costs of healthcare, concerns about seeing a doctor because of immigration status, or no healthcare coverage/insurance.

We dichotomized each index as less than or equal to the median value for statistical models: more versus less potential exposure risk, more versus less susceptible to COVID-19 complications, and more versus less difficulty with access to care. The indices (exposure, susceptibility, and access) came from baseline recruitment surveys.

### COVID-19 Outcomes by Hospitalization and Seroconversion

We examined the association of potential exposure, susceptibility, or access to care with 2 COVID-19 outcomes: COVID-19 hospitalization and observed seroconversion. We defined COVID-19 hospitalization as a self-report of hospitalization for any COVID-19‒like symptoms from baseline through the eighth follow-up assessment (V0‒V8, March 2020‒October 2021). We asked the following question: “Since you completed your last survey on DD/MM/YYYY, were you hospitalized for any of these symptoms?” We dichotomized outcome as yes or no and classified persons who reported do not know/not sure as no.

The procedure for at-home specimen collection for serologic testing has been reported (*20*). In brief, all participants were invited to participate in serologic testing by using an at-home self-collected dried blood spot specimen collection kit during May‒August 2020 (period 1) and November 2020‒January 2021 (period 2). All dried blood spot specimens were tested by the study laboratory for total antibodies by using the Platelia Test (Bio-Rad Laboratories, https://www.bio-rad.com) for IgA, IgM, and IgG, which targets the SARS-CoV-2 nucleocapsid protein (*21*). A total of 4,233 (63%) participants underwent serologic testing in period 1 and 3,884 (58%) in period 2. Of the 4,510 participants who tested at least once, 3,605 (80%) tested at both time points (*20*). Among those persons who had 2 total antibody tests, an observed seroconversion was defined as a negative total antibody test result in period 1, followed by a positive total antibody test result in period 2 (n = 3,422).

### Confounders

We treated age, sex, presence of children in the household, income, education, or employment as possible confounders of the hypothesized exposure-outcome relationships. We identified confounders a priori based on directed acyclic graph framework (Appendix Figures 1–3) (*22*) and identified the minimum sufficient adjustment set for estimating the total effect of a given exposure on outcomes.

### Statistical Analysis

We used descriptive statistics to examine participant demographics and indices reflecting potential SARS-CoV-2 exposure, susceptibility, and access to healthcare stratified by race/ethnicity. We assessed differences between groups by using the χ^2^ or Kruskal-Wallis test as appropriate.

We used a logistic regression model to assess the association between each index and outcomes of interest: COVID-19-hospitalization or seroconversion. We separately modeled each exposure-outcome relationship. When potential SARS-CoV-2 exposure was the explanatory index of interest, we adjusted for age, presence of children in the household, employment, income, race/ethnicity. When susceptibility was the explanatory index of interest, we adjusted for employment, income, race/ethnicity, and we did not adjust for age because age was used to create the susceptibility summative score. When access was the explanatory index of interest, we adjusted for age, employment, sex, income, race/ethnicity.

We assessed whether the effect of each index on COVID-19 outcomes was modified by race/ethnicity. We assessed EMM on the additive scale and present the relative excess risk caused by interaction (RERI) (*23*,*24*). Because EMM on the additive scale indicates whether the effect of an exposure is different in 1 subpopulation relative to another, assessing the additive interaction is useful for identifying the specific population for whom public health interventions will have the greatest effect (*23*,*24*). We collapsed the race variable to White non-Hispanic versus Hispanic/Latino/a and Black non-Hispanic for assessment of EMM.

We conducted logistic regression models with SAS version 9.4 (https://www.sas.com). We generated 95% CIs for RERI by using the spreadsheet tool reported by Knol and VanderWeele. (*23*).

## Results

This analysis used data for 6,740 persons enrolled into prospective follow-up for analyses assessing the hospitalization outcome reported through October 20, 2021. Among the full cohort, 19% (n = 1,308) identified as Hispanic/Latino/a ethnicity, 13% (n = 899) as Black non-Hispanic, 7% (n = 465) as Asian/Pacific Islander non-Hispanic, 57% (n = 3,846) as White non-Hispanic, and 3% (n = 222) as other non-Hispanic race (Table 1). Hispanic/Latino/a (mean +SD age 35 +13 years), Black non-Hispanic (mean +SD age 35 +13 years), or Asian/Pacific Islander non-Hispanic participants (mean +SD age 33 +12 years) were younger on average than White non-Hispanic participants (mean +SD age 45 +16). More than half (52%) of the cohort were women. More than half (57%) of the cohort had a college-level education, and the proportion with a college-education was highest among Asian/Pacific Islander non-Hispanic (69%) and lowest among Black non-Hispanic participants (33%).

**Table 1:** Demographic and socioeconomic characteristics of communities, households, and SARS-CoV-2 epidemiology for Chasing COVID study participants, stratified by race and ethnicity, United States, March 28‒April 20, 2020*

Variable	Total	Hispanic or Latino/a	Black non-Hispanic	Asian/Pacific Islander non-Hispanic	White non-Hispanic	Other non-Hispanic	p value
Total	6,740 (100.00)	1,308 (19.41)	899 (13.33)	465 (6.90)	3,846 (57.06)	222 (3.30)	
Age, y							<0.001
Mean (SD)	40.61 (15.28)	35.19 (13.33)	35.31 (12.80)	32.73 (11.95)	44.64 (15.54)	40.74 (14.06)	
Median (IQR)	37 (29‒51)	33 (25‒42)	32 (26‒42)	30 (24‒39)	42 (32‒57)	39 (29‒49)	
Sex							<0.001
M	3,043 (45.15)	568 (43.43)	411 (45.72)	195 (41.94)	1,762 (45.81)	107 (48.2)	
F	3,526 (52.31)	718 (54.89)	468 (52.06)	260 (55.91)	1,983 (51.56)	97 (43.69)	
Nonbinary	171 (2.54)	22 (1.68)	20 (2.22)	10 (2.15)	101 (2.63)	18 (8.11)	
Education							<0.001
<12th grade	123 (1.82)	34 (2.6)	25 (2.78)	9 (1.94)	54 (1.4)	1 (0.45)	
12th grade/GED	875 (12.98)	282 (21.56)	191 (21.25)	36 (7.74)	330 (8.58)	36 (16.22)	
College, 1–3 y	1,889 (28.03)	436 (33.33)	385 (42.83)	100 (21.51)	894 (23.24)	74 (33.33)	
College, >4 y	3,853 (57.17)	556 (42.51)	298 (33.15)	320 (68.82)	2,568 (66.77)	111 (50.00)	
Employment status							<0.001
Employed	4,247 (63.01)	811 (62)	587 (65.29)	267 (57.42)	2,443 (63.52)	139 (62.61)	
Out of work	830 (12.31)	206 (15.75)	131 (14.57)	55 (11.83)	402 (10.45)	36 (16.22)	
Other	1,663 (24.67)	291 (22.25)	181 (20.13)	143 (30.75)	1,001 (26.03)	47 (21.17)	
Income							<0.001
<$35,000	1,969 (29.21)	468 (35.78)	415 (46.16)	111 (23.87)	878 (22.83)	97 (43.69)	
$35,000‒$49,999	753 (11.17)	180 (13.76)	111 (12.35)	39 (8.39)	394 (10.24)	29 (13.06)	
$50,000‒$69,999	959 (14.23)	210 (16.06)	148 (16.46)	58 (12.47)	520 (13.52)	23 (10.36)	
$70,000‒$99,999	1,058 (15.70)	179 (13.69)	82 (9.12)	88 (18.92)	683 (17.76)	26 (11.71)	
>$100,000	1,793 (26.60)	228 (17.43)	115 (12.79)	142 (30.54)	1,266 (32.92)	42 (18.92)	
Do not know	208 (3.09)	43 (3.29)	28 (3.11)	27 (5.81)	105 (2.73)	5 (2.25)	
Children <18 y of age							<0.001
No	4,564 (67.72)	692 (52.91)	534 (59.40)	314 (67.53)	2,879 (74.86)	145 (65.32)	
Yes	2,176 (32.28)	616 (47.09)	365 (40.60)	151 (32.47)	967 (25.14)	77 (34.68)	

*Values are no. (%) unless otherwise indicated. Chasing COVID, Communities, Households, and SARS-CoV-2 Epidemiology COVID Cohort Study; GED, general educational development; IQR, interquartile range.

For seroconversion analyses, we used a subset of 3,422 participants seronegative in May‒September 2020 who tested again during November 2020‒January 2021. Compared with the full cohort, the subset of testers had more White non-Hispanic participants (57% vs. 67%), was older (mean age 44 years vs. 41 years), and had higher educational attainment (57% vs. 67% with at least a college education) (Appendix Table 1).

### Potential SARS-CoV-2 Exposure Risk by Built-Environment and Work-Related Ability to Social Distance

For built-environment measures of exposure (Table 2), greater percentages of Hispanic/Latino/a, Black non-Hispanic, and Asian/Pacific Islander non-Hispanic participants lived in urban areas and in multiunit dwellings compared with White non-Hispanic participants. A greater percentage of Hispanic/Latino/a and Black non-Hispanic participants were unable to avoid public transportation compared with Asian/Pacific Islander non-Hispanic and White non-Hispanic participants. For work-related measures, the percentage of participants with less ability to social distance was generally highest among Black non-Hispanic participants and lowest among White non-Hispanic participants. A greater percentage of Black non-Hispanic participants than White non-Hispanic participants who were employed reported that they were unable to work from home and could lose their job if unable to go to work. The percentage with more exposure risk was highest among Black non-Hispanic participants (51%) and Hispanic/Latino/a participants (46%) and lowest among Asian/Pacific Islander non-Hispanic participants (36%) and White non-Hispanic participants (33%). All reported differences were statistically significant.

**Table 2:** Measures of potential SARS-CoV-2 exposure, susceptibility to COVID-19 complications, and access to care for Chasing COVID study participants, stratified by race/ethnicity, United States, March 28‒April 20, 2020*

Variable	Overall, n = 6,740	Hispanic, n = 1,308	Black non-Hispanic, n = 899	Asian/Pacific Islander, n = 465	White non-Hispanic, n = 3,846	Other, n = 222	p value†
Measures of potential exposure: inability to impose social distance							
Built environment measures							
Living in urban area	2,820 (41.84)	563 (43.04)	414 (46.05)	225 (48.39)	1,528 (39.73)	90 (40.54)	0.001
Living in multidwelling building	2,636 (39.11)	505 (38.61)	416 (46.27)	202 (43.44)	1,420 (36.92)	93 (41.89)	<0.001
Ability to avoid public transportation	629 (9.33)	155 (11.85)	153 (17.02)	27 (5.81)	266 (6.92)	28 (12.61)	<0.001
Median no. measures (IQR)	1 (0‒2)	1 (0‒2)	1 (0‒2)	1 (0‒2)	1 (0‒1)	1 (0‒2)	<0.001
Work-related measures							
Unable to work from home	1,825 (27.08)	398 (30.43)	299 (33.26)	102 (21.94)	952 (24.75)	74 (33.33)	<0.001
Will not get paid if at home	1,585 (23.52)	364 (27.83)	263 (29.25)	110 (23.66)	781 (20.31)	67 (30.18)	<0.001
Does not have sick leave	1,754 (26.02)	375 (28.67)	300 (33.37)	115 (24.73)	888 (23.09)	76 (34.23)	<0.001
Could lose job or business if unable to go to work	1,542 (22.88)	372 (28.44)	285 (31.70)	95 (20.43)	723 (18.80)	67 (30.18)	<0.001
Job can only be done in workplace	2,023 (30.01)	456 (34.86)	331 (36.82)	121 (26.02)	1,049 (27.28)	66 (29.73)	<0.001
Essential worker	588 (8.72)	116 (8.87)	84 (9.34)	38 (8.17)	329 (8.55)	21 (9.46)	0.92
Median no. measures (IQR)	1 (02)	1 (0‒3)	2 (0‒3)	1 (0‒2)	0 (0‒2)	1 (0‒3)	<0.001
Median no. built-environment and work-related measures (IQR)	2 (1‒3)	2 (1‒4)	3 (1‒4)	2 (1‒3)	2 (1‒3)	2 (1‒4)	<0.001
More potential exposure risk: index >2	2,596 (38.52)	601 (45.95)	462 (51.39)	166 (35.70)	1,272 (33.07)	95 (42.79)	<0.001
Measures of susceptibility							
Age >60 y	1,027 (15.24)	76 (5.81)	54 (6.01)	22 (4.73)	847 (22.02)	28 (12.61)	<0.001
Chronic lung disease	194 (2.88)	35 (2.68)	18 (2.00)	8 (1.72)	120 (3.12)	13 (5.86)	0.01
Asthma (current)	752 (11.16)	143 (10.93)	108 (12.01)	34 (7.31)	429 (11.15)	38 (17.12)	<0.01
T2 diabetes	490 (7.27)	129 (9.86)	66 (7.34)	15 (3.23)	259 (6.73)	21 (9.46)	<0.001
Serious heart condition	1,542 (22.88)	271 (20.72)	240 (26.7)	42 (9.03)	938 (24.39)	51 (22.97)	<0.001
Kidney disease	105 (1.56)	23 (1.76)	8 (0.89)	1 (0.22)	69 (1.79)	4 (1.8)	0.04
Immunocompromised	180 (2.67)	27 (2.06)	13 (1.45)	6 (1.29)	126 (3.28)	8 (3.60)	<0.01
HIV	268 (3.98)	49 (3.75)	63 (7.01)	5 (1.08)	143 (3.72)	8 (3.60)	<0.001
Daily smoker	997 (14.79)	228 (17.43)	208 (23.14)	30 (6.45)	470 (12.22)	61 (27.48)	<0.001
Median no. measures (IQR)	1 (0‒1)	0 (0‒1)	1 (0‒1)	0 (0‒1)	1 (0‒1)	1 (0‒2)	<0.001
More susceptible index >1	1,453 (21.56)	238 (18.20)	202 (22.47)	30 (6.45)	924 (24.02)	59 (26.58)	<0.001
Measures of healthcare access							
Does not have 1 person as doctor	1,960 (29.08)	464 (35.47)	330 (36.71)	156 (33.55)	921 (23.95)	89 (40.09)	<0.001
Did not see doctor due to cost	1,277 (18.95)	327 (25.00)	221 (24.58)	84 (18.06)	591 (15.37)	54 (24.32)	<0.001
Did not see doctor due to immigration	288 (4.27)	124 (9.48)	66 (7.34)	16 (3.44)	71 (1.85)	11 (4.95)	<0.001
No insurance	1,172 (17.39)	347 (26.53)	242 (26.92)	87 (18.71)	450 (11.7)	46 (20.72)	<0.001
Median no. measures (IQR)	0 (0‒1)	1 (0‒2)	1 (0‒2)	0 (0‒1)	0 (0‒1)	1 (0‒1)	<0.001
More barriers to access: index >0	3,050 (45.25)	749 (57.26)	510 (56.73)	231 (49.68)	1,430 (37.18)	231 (49.68)	<0.001

*Values are no. (%) responding yes unless otherwise indicated. Chasing COVID, Communities, Households, and SARS-CoV-2 Epidemiology COVID Cohort Study; IQR, interquartile range. †Based on the χ^2^ test for categorical data or the Kruskal-Wallis test for summative indices.

### Susceptibility

Asian/Pacific Islander non-Hispanic participants generally had the lowest frequency of individual metrics of COVID-19 susceptibility. Hispanic/Latino/a, Black, and White non-Hispanic participants were more likely to report a serious heart condition and current asthma than were Asian/Pacific Islander non-Hispanic participants (p<0.01). Hispanic/Latino/a and Black non-Hispanic participants were more likely to report daily smoking than were Asian/Pacific Islander non-Hispanic or White non-Hispanic participants (p<0.001). The percentage more susceptible was higher for White non-Hispanic (24%), Black non-Hispanic (23%), and Hispanic/Latino/a (18%) participants than for Asian/Pacific Islander non-Hispanic participants (7%) (p<0.001).

### Healthcare Access

Hispanic/Latino/a, Black non-Hispanic, and Asian/Pacific Islander non-Hispanic participants were more likely than White non-Hispanic participants to report having no primary care doctor, not seeing a doctor because of cost, not seeing a doctor because of immigration status, and not having insurance (p<0.001). The percentage reporting more difficulty with access to healthcare was higher among Hispanic/Latino/a (57%), Black non-Hispanic (57%), and Asian/Pacific Islander non-Hispanic participants (50%) than among White non-Hispanic participants (37%) (p<0.001). Trends in potential exposure, susceptibility, and healthcare access in the subset of testers mirrored trends in the full cohort (Appendix Table 2).

### Association of Potential Exposure, Susceptibility, and Access to Care with COVID-19 Outcomes

Approximately 5% (n = 161/3,422) of participants seroconverted, and 6% (n = 401/6,070) were hospitalized (Table 3). In models adjusted for sociodemographics including age, participants who had more (versus less) exposure risk had greater odds of seroconversion (adjusted odds ratio [aOR] 1.64, 95% CI 1.17–2.30) and hospitalization (aOR 1.70, 95% CI 1.37–2.12) (Table 3). Neither susceptibility nor access to care was associated with seroconversion. However, participants who had more (versus less) susceptibility and those who had more (versus less) difficulty with healthcare access had greater odds of hospitalization (aOR_susceptibility_ 2.35, 95% CI 1.88–2.92 and aOR_access_ 2.28, 95% CI 1.81–2.87).

**Table 3:** Effects of potential SARS-CoV-2 exposure, susceptibility to COVID-19 complications, and access to healthcare on odds of seroconversion (n = 3,422) and hospitalization (n = 6,740) for Chasing COVID study participants, United States, March 28‒April 20, 2020*

Variable	Seroconversion				Hospitalization		
	No. (%)	OR (95% CI)	aOR (95% CI)		No. (%)	OR (95% CI)	aOR (95% CI)
Overall	161 (4.70)				401 (5.95)		
Potential exposure†							
Less exposure risk	86 (3.73)	Referent	Referent		178 (4.30)	Referent	Referent
More exposure risk	75 (6.73)	1.86 (1.35‒2.56)	1.64 (1.17‒2.30)		223 (8.59)	2.09 (1.71‒ 2.57)	1.70 (1.37‒2.12)
Susceptibility‡							
Less susceptible	130 (4.95)	Referent	Referent		258 (4.88)	Referent	Referent
More susceptible	31 (3.90)	0.78 (0.52‒1.16)	0.82 (0.54‒1.24)		143 (9.84)	2.13 (1.72‒ 2.63)	2.35 (1.88‒2.92)
Access to healthcare§							
Less barriers	93 (4.21)	Referent	Referent		130 (3.52)	Referent	Referent
More barriers	68 (5.61)	1.35 (0.98‒1.86)	1.22 (0.87‒1.71)		271 (8.89)	2.67 (2.15‒3.31)	2.28 (1.81‒2.87)

*aOR, adjusted OR; Chasing COVID, Communities, Households, and SARS-CoV-2 Epidemiology COVID Cohort Study; OR, odds ratio. †Model adjusted for age, presence of children in the household, employment, income, race/ethnicity. ‡Model adjusted for employment, income, race/ethnicity. §Model adjusted for age, employment, sex, income, race/ethnicity.

### EMM by Race/Ethnicity

Hispanic/Latino/a and Black non-Hispanic participants were more likely to seroconvert or to be hospitalized for COVID-19 than Asian/Pacific Islander non-Hispanic or White non-Hispanic participants (seroconversion 7% and 6% vs. 4% and 3%, respectively [p<0.01]; hospitalization 8%, and 9% vs. 5% and 3%, respectively [p<0.001]) (Appendix Table 3). For the seroconversion outcome, we saw no evidence of EMM by race/ethnicity (Appendix Table 4). For the hospitalization outcome, we saw evidence of EMM by race/ethnicity for the susceptibility index (RERI 1.75; p<0.01), meaning that Hispanic/Latino/a or Black non-Hispanic participants who had a high score on the susceptibility index were at disproportionately higher odds of COVID hospitalization compared with White non-Hispanic participants. The odds of COVID hospitalization were 2.70 (95% CI 1.95–3.72) for Hispanic/Latino/a or Black non-Hispanic participants and 2.14 (95% CI 1.55–2.14) for White non-Hispanic participants. In contrast, there was no evidence of EMM by race/ethnicity for potential SARS-CoV-2 exposure or healthcare access indices with hospitalization (Table 4).

**Table 4:** Modification of the association between race/ethnicity and hospitalization by potential SARS-CoV-2 exposure, susceptibility, and healthcare access for Chasing COVID study participants (n = 6,053), United States, March 28‒April 20, 2020*

Variable	White non-Hispanic			Hispanic/Latino/a or Black non-Hispanic		aOR (95% CI) within exposed strata, Hispanic/Latino/a or Black non-Hispanic versus White
	No. hospitalized/denominator (%)	aOR (95% CI)		No. hospitalized/denominator (%)	aOR (95% CI)	
Measure of potential exposure†						
Less exposure risk	99/2,574 (3.85)	Referent		63/1,144 (5.51)	1.10 (0.78‒1.55)	1.10 (0.78‒1.55)
More exposure risk	86/1272 (6.76)	1.57 (1.16‒2.15)		123/1,063 (11.57)	2.30 (1.69‒3.13)	1.46 (1.08‒1.97)
Less versus more within strata		1.57 (1.16‒2.15)			2.09 (1.51‒2.89)	
p value		p<0.01			p<0.001	
RERI (95% CI): measure of interaction on the additive scale					0.63 (−0.01 to 1.26)	
p value					p = 0.05	
Susceptibility‡						
Less susceptible	119/2,922 (4.07)	Referent		118/1,767 (6.68)	1.71 (1.30‒2.23)	1.71 (1.30‒2.23)
More susceptible	66/924 (7.14)	2.14 (1.55‒2.94)		68/440 (15.45)	4.60 (3.33‒6.36)	2.15 (1.49‒3.10)
More versus less within strata		2.14 (1.55‒2.94)			2.70 (1.95‒3.72)	
p value		p<0.001			p<0.001	
RERI (95% CI): measure of interaction on the additive scale					1.75 (0.39‒3.11)	
p value					p = 0.001	
Healthcare access§						
Less barriers to access	78/2,416 (3.23)	Referent		44/948 (4.64)	1.37 (0.93‒2.03)	1.37 (0.93‒2.03)
More barriers to access	107/1,430 (7.48)	2.23 (1.63‒3.04)		142/1,259 (11.28)	3.41 (2.47‒4.71)	1.53 (1.17‒2.01)
Less versus more within strata of race/ethnicity		2.23 (1.63‒3.04)			2.48 (1.74‒3.54)	
p value		p<0.001			p<0.001	
RERI (95% CI): measure of interaction on the additive scale					0.81 (−0.06 to 1.69)	
p value					p = 0.07	

*aOR, adjusted OR; Chasing COVID, Communities, Households, and SARS-CoV-2 Epidemiology COVID Cohort Study; OR, odds ratio: RERI, relative excess risk caused by interaction. †Model adjusted for age, presence of children in the household, employment, income, race/ethnicity. ‡Model adjusted for employment, income, race/ethnicity. §Model adjusted for age, employment, sex, income, race/ethnicity.

## Discussion

Our study confirms the existence of major racial and ethnic differences in potential SARS-CoV-2 exposure risk, susceptibility to COVID-19 complications, and access to healthcare within a large US national cohort. The percentage of those with more potential exposure risk and more difficulty with healthcare access was higher among Black non-Hispanic, Hispanic/Latino/a, and Asian/Pacific Islander non-Hispanic participants than among White non-Hispanic participants. Greater potential exposure, as measured by reduced ability to social distance, increased the odds of seroconversion by 64% and hospitalization by 70%. Greater underlying susceptibility and difficulty with access to care increased the odds of hospitalization by 128% to 135%.

Many researchers have hypothesized that social determinants have driven disparities in the effect of the COVID-19 pandemic, either directly or indirectly, because of occupation, living and working conditions, health-related behaviors, comorbidities, and immune functioning (*6*,*8*,*11*). However, the influence of social determinants on COVID-19 outcomes is understudied, and existing research has largely characterized social determinants by using geography and race/ethnicity as proxies (*25**–**30*). For example, US counties that have a higher proportion of Black or Hispanic population or of adults with less than a high school diploma had disproportionately higher numbers of COVID-19 cases (*29*). Using data from the American Community Survey to characterize socioeconomic vulnerability at the neighborhood level, ecologic analyses have demonstrated that increasing levels of socioeconomic vulnerability were associated with gaps in COVID-19 testing coverage in Massachusetts and COVID-19 deaths in Chicago, Illinois (*25*,*30*). Although useful, such approaches might mask the extent of COVID-19 disparities and the influence of social determinants at the individual level. We are aware of 1 study that included individual-level social indicators to assess COVID-19 outcomes (*31*).

Hispanic ethnicity, inability to shelter in place and maintain income, frontline service work, unemployment, and household income <$50,000 increased the risk for COVID-19 infection among residents and workers located in small community within San Francisco, California (*31*). We provide empirical evidence to support the conceptual model of Blumenshine et al. (*11*) in the context of the COVID-19 pandemic. Differences in social factors contribute to disparities in SARS-CoV-2 exposure, susceptibility to illness given infection, and access to care. Furthermore, reduced ability to social distance was positively associated with seroconversion and hospitalization, and increased susceptibility to COVID-19 complications and poor access to healthcare were positively associated with hospitalization.

We did not observe an association between seroconversion and susceptibility or access to care. The null finding is unsurprising given susceptibility to complications and access to care would be expected to influence illness after infection. Primary and secondary prevention efforts should address potential social disparities in exposure, COVID-19 vaccination, and access to care/treatment.

Our finding that Hispanic or Latino/a and Black non-Hispanic participants had more potential exposure risk and more difficulty with healthcare access than White non-Hispanic participants is consistent with other research showing a disproportionate burden of COVID-19 infections, complications, and deaths among racial and ethnic minorities (*3*,*5*,*8*,*27*,*32**–**37*). The positive additive interaction observed between racial and ethnic minority group status and susceptibility to more severe COVID-19 outcomes with hospitalization is especially concerning. We did not observe evidence of EMM by race/ethnicity in terms of the COVID exposure index or the healthcare access index. Recommendations for and discussions about social distancing fail to account for the reality of differential ability to adopt and benefit from these approaches, creating inequities in health outcomes. Longstanding social and health inequities contribute to susceptibility among Hispanic/Latino/a and Black non-Hispanic persons, and susceptibility is also influenced by lower healthcare access. Mitigation strategies and messaging should intensify focus on Hispanic/Latino/a and Black non-Hispanic persons who have conditions that increase risk for COVID-19 illness and death and incorporate tailored, culturally appropriate communication.

The first limitation of our study is that unmeasured confounding might effect exposure-outcome effect measures. We did not control for the time-varying nature of vaccination status or mask use because we considered these variables to lie on the causal pathway (Appendix Figures 1–3). We also did not address the possibility of joint effects of the indices. 

Second, participants might not have completed every survey or serologic test, which would affect outcome measurement. Enrollment into the prospective cohort required 2 study interactions (i.e., completing the baseline survey and a second survey or the first serologic test). There was no missing data for the exposure measures or confounder measures because these measures were derived from the enrollment assessment, and participants had >1 opportunity to contribute data to the hospitalization outcome. Furthermore, cohort participation was high. A total of 58% (n = 3,913) of participants completed all 8 surveys included in this analysis, whereas 16% (n = 1,073) completed only 1 survey. Analyses of seroconversion were restricted to the population of persons who were seronegative at survey 1 and had 2 serologic tests (n = 3,422) (Appendix Table 1).

Third, measurement error and reporting bias might be a concern for measures of the exposure indices and hospitalizations. Although the indices have been previously used in national surveys conducted during the influenza A(H1N1) pandemic, those indices were proxies for exposure, seroconversion, and access to care and did not fully capture all aspects of these constructs (e.g., health literacy). This survey was launched in March 2020, when access to SARS-CoV-2 tests was severely limited and persons were hospitalized on the basis of symptoms. Therefore, we asked participants about hospitalization caused by COVID-19‒like symptoms, rather than COVID-19 specifically. Accordingly, we might have inadvertently included some non–COVID-19 hospitalizations, particularly later in the pandemic.

Fourth, small numbers prevented us from assessing effect modification for each race/ethnicity group. We ran models comparing all race/ethnicities (Hispanic/Latino/a, Black non-Hispanic, Asian/Pacific Islander non-Hispanic, or other non-Hispanic) versus White non-Hispanic, and the results were similar to the models comparing Hispanic/Latino/a and Black non-Hispanic versus White non-Hispanic. Last, because our study is not a probability or population-based sample, findings might not be generalizable to all of the US population.

There have been increasing calls for research to better capture and report on socioeconomic determinants of COVID-19 outcomes alongside race/ethnicity to identify populations that might experience a disproportionate burden of risk or ability to benefit from pandemic mitigation strategies (*10*,*12*). We observed major racial/ethnic inequities in ability to social distance as a measure of potential SARS-CoV-2 exposure, susceptibility to COVID-19 complications, and access to healthcare in our national cohort. Future pandemic mitigation strategies should account for the contribution of social factors to racial and ethnic disparities in pathogen exposure, susceptibility to disease, and healthcare access.

Appendix Additional information on racial/ethnic disparities in exposure, disease susceptibility, and clinical outcomes during COVID-19 pandemic in national cohort of adults, United States.
