# Supplementary material for: Racial/Ethnic Disparities in Exposure, Disease Susceptibility, and Clinical Outcomes during COVID-19 Pandemic in National Cohort of Adults, United States
Source: Emerg Infect Dis. 2022 Nov;28(11):2171–80. doi: 10.3201/eid2811.220072 (PMC9622253; doi:10.3201/eid2811.220072)
Supplement: Appendix — Additional information on racial/ethnic disparities in exposure, disease susceptibility, and clinical outcomes during COVID-19 pandemic in national cohort of adults, United States. [file 22-0072-Techapp-s1.pdf]

Article DOI: https://doi.org/10.3201/eid2811.220057

Racial/Ethnic Disparities in Exposure, Disease Susceptibility, and Clinical Outcomes during COVID-19 Pandemic in National Cohort of Adults, United States

Appendix

Appendix Table 1. Demographic and Socioeconomic Characteristics of the Chasing COVID Cohort (C3) Participants who were Seronegative and Retested - United States, Enrolled from March 28 - April 20, 2020 (N = 3,422)*

Characteristic	Total	Hispanic	Black NH	Asian\Pacific Islander NH	White NH	Other NH	p value†
Total, n (%)	3,422 (100.00)	500 (14.61)	279 (8.15)	219 (6.4)	2,312 (67.56)	112 (3.28)	
Age							<0.001
Mean (SD)	44 (15.16)	39.51 (13.13)	40.5 (12.90)	33 (13.18)	46.62 (15.43)	44.33 (13.57)	
Median (IQR)	42 (32, 56)	37 (30, 48)	37 (30, 51)	33 (26, 41)	45 (34, 60)	43 (35, 53)	
Age Category, n (%)							<0.001
18–29	617 (18.03)	120 (24.00)	57 (20.43)	84 (38.36)	342 (14.79)	14 (12.50)	
30–39	934 (27.29)	166 (33.20)	100 (35.84)	76 (34.70)	566 (24.48)	26 (23.21)	
40–49	654 (19.11)	104 (20.80)	50 (17.92)	30 (13.70)	430 (18.60)	40 (35.71)	
50–59	514 (15.02)	62 (12.40)	40 (14.34)	11 (5.02)	388 (16.78)	13 (11.61)	
60+	703 (20.54)	48 (9.60)	32 (11.47)	18 (8.22)	586 (25.35)	19 (16.96)	
Sex, n (%)							<0.001
M	1,516 (44.30)	219 (43.80)	124 (44.44)	88 (40.18)	1,033 (44.68)	52 (46.43)	
F	1,810 (52.89)	267 (53.40)	147 (52.69)	128 (58.45)	1,221 (52.81)	47 (41.96)	
Non-Binary	96 (2.81)	14 (2.80)	8 (2.87)	3 (1.37)	58 (2.51)	13 (11.61)	
Education, n (%)							<0.001
Less than 12th grade	37 (1.08)	10 (2.00)	2 (0.72)	2 (0.91)	22 (0.95)	1 (0.89)	
12th grade/GED	278 (8.12)	71 (14.20)	33 (11.83)	6 (2.74)	155 (6.70)	13 (11.61)	
Some college (1–3 y)	815 (23.82)	151 (30.20)	112 (40.14)	38 (17.35)	488 (21.11)	26 (23.21)	
College (≥4 years)	2,292 (66.98)	268 (53.60)	132 (47.31)	173 (79.00)	1,647 (71.24)	72 (64.29)	
Employment Status, n (%)							<0.001
Employed	2,102 (61.43)	284 (56.80)	181 (64.87)	131 (59.82)	1,436 (62.11)	70 (62.50)	
Out of Work	402 (11.75)	88 (17.60)	42 (15.05)	26 (11.87)	229 (9.90)	17 (15.18)	
Other	918 (26.83)	128 (25.60)	56 (20.07)	62 (28.31)	647 (27.98)	25 (22.32)	
Income, n (%)							<0.001
Less than $35,000	870 (25.42)	174 (34.80)	113 (40.50)	47 (21.46)	494 (21.37)	42 (37.50)	
$35,000 - $49,999	383 (11.19)	77 (15.40)	43 (15.41)	16 (7.31)	231 (9.99)	16 (14.29)	
$50,000 - $69,999	505 (14.76)	75 (15.00)	56 (20.07)	30 (13.70)	333 (14.40)	11 (9.82)	
$70,000 - $99,999	592 (17.30)	73 (14.60)	30 (10.75)	43 (19.63)	436 (18.86)	10 (8.93)	
$100,000+	993 (29.02)	90 (18.00)	33 (11.83)	72 (32.88)	768 (33.22)	30 (26.79)	
Don't know	79 (2.31)	11 (2.20)	4 (1.43)	11 (5.02)	50 (2.16)	3 (2.68)	
Any Children <18, n (%)							<0.001
No	2,596 (75.86)	326 (65.20)	183 (65.59)	154 (70.32)	1,853 (80.15)	80 (71.43)	
Yes	826 (24.14)	174 (34.80)	96 (34.41)	65 (29.68)	459 (19.85)	32 (28.57)	

*IQR: Interquartile Range, NH: non-Hispanic, n: number, SD: standard deviation, U.S.: United States, and %: percentage.
†p value based on the Kruskal-Wallis test for means and Chi square for frequencies.

Appendix Table 2. Measures of Exposure, Susceptibility and Access to Care – Among Participants who were Seronegative and Retested (N = 3,422)*

Category	Overall (N = 6,740)	Hispanic (N = 1,308)	Black NH (N = 899)	Asian\Pacific Islander (N = 465)	White NH (N = 3,846)	Other (N = 222)	p value†
Measures of potential exposure: inability to impose social distance							
Built environment measures							
Living in an urban area	1,430 (41.79)	219 (43.80)	140 (50.18)	116 (52.97)	906 (39.19)	49 (43.75)	<0.001
Living in an multidwelling building	1,348 (39.39)	206 (41.20)	144 (51.61)	93 (42.47)	859 (37.15)	46 (41.07)	<0.001
Ability to avoid public transportation	218 (6.37)	38 (7.60)	37 (13.26)	7 (3.20)	128 (5.54)	8 (7.14)	<0.001
Sum built-environment							
Median (IQR)	1 (0,2)	1 (0,2)	1 (0,2)	1 (0,2)	1 (0,1)	1 (0,2)	<0.001
Work-related measures							
Not able to work from home	849 (24.81)	134 (26.80)	87 (31.18)	42 (19.18)	554 (23.96)	32 (28.57)	0.01
Will not get paid if at home	632 (18.47)	104 (20.80)	70 (25.09)	38 (17.35)	398 (17.21)	22 (19.64)	0.01
Does not have sick leave	746 (21.80)	116 (23.20)	82 (29.39)	46 (21.00)	474 (20.50)	28 (25.00)	0.01
Could lose job or business if unable to go to work	597 (17.45)	113 (22.60)	69 (24.73)	38 (17.35)	353 (15.27)	24 (21.43)	<0.001
Job can only be done in workplace	911 (26.62)	157 (31.40)	98 (35.13)	50 (22.83)	577 (24.96)	29 (25.89)	<0.01
Essential worker	282 (8.24)	34 (6.80)	32 (11.47)	15 (6.85)	189 (8.17)	12 (10.71)	0.15
Sum work-related measures							<0.001
Median (IQR)	1 (0,2)	1 (0,3)	2 (0,3)	1 (0,2)	0 (0,2)	1 (0,3)	
Sum built-environment and work-related measures							
Median (IQR)	2 (1,3)	2 (1,4)	3 (1,4)	2 (1,3)	2 (1,3)	2 (1,4)	<0.001
More potential exposure risk: index >2	1,115 (32.58)	189 (37.80)	130 (46.59)	69 (31.51)	687 (29.71)	40 (35.71)	<0.001
Measures of Susceptibility							
Age 60+	703 (20.54)	48 (9.60)	32 (11.47)	18 (8.22)	586 (25.35)	19 (16.96)	<0.001
Chronic Lung Disease	103 (3.01)	17 (3.40)	9 (3.23)	5 (2.28)	64 (2.77)	8 (7.14)	0.10
Asthma (current)	389 (11.37)	62 (12.40)	35 (12.54)	10 (4.57)	261 (11.29)	21 (18.75)	<0.01
T2 Diabetes	231 (6.75)	44 (8.80)	29 (10.39)	5 (2.28)	140 (6.06)	13 (11.61)	<0.01
Serious heart condition	865 (25.28)	115 (23.00)	89 (31.90)	20 (9.13)	613 (26.51)	28 (25.00)	<0.001
Kidney disease	50 (1.46)	7 (1.40)	2 (0.72)	1 (0.46)	39 (1.69)	1 (0.89)	0.45
Immunocompromised	108 (3.16)	13 (2.60)	7 (2.51)	3 (1.37)	80 (3.46)	5 (4.46)	0.34
HIV	161 (4.70)	25 (5.00)	30 (10.75)	3 (1.37)	97 (4.20)	6 (5.36)	<0.001
Daily smoker	343 (10.02)	58 (11.60)	49 (17.56)	8 (3.65)	202 (8.74)	26 (23.21)	<0.001
Sum susceptibility measures							
Median (IQR)	1 (0, 1)	0 (0,1)	1 (0,1)	0 (0,1)	1 (0,1)	1 (0,2)	<0.001
More susceptible: index >1	795 (23.23)	98 (19.60)	78 (27.96)	15 (6.85)	570 (24.65)	34 (30.36)	<0.001
Measures of healthcare access							
Does not have one person as doctor	808 (23.61)	145 (29.00)	74 (26.52)	69 (31.51)	483 (20.89)	37 (33.04)	<0.001
Did not see doctor due to cost	510 (14.90)	94 (18.80)	58 (20.79)	41 (18.72)	295 (12.76)	22 (19.64)	<0.001
Did not see doctor due to immigration	27 (0.79)	13 (2.60)	4 (1.43)	5 (2.28)	2 (0.09)	3 (2.68)	<0.001
No insurance	384 (11.22)	88 (17.60)	56 (20.07)	27 (12.33)	195 (8.43)	18 (16.07)	<0.001
Sum healthcare access measures							
Median (IQR)	0 (0.1)	1 (0,2)	1 (0,2)	0 (0,1)	0 (0,1)	1 (0,1)	<0.001
More difficulty with access: index >0	1,213 (35.45)	217 (43.40)	121 (43.37)	97 (44.29)	726 (31.40)	52 (46.43)	<0.001

*IQR: interquartile range, SD: standard deviation.
†p value statistic is based on the chi-square test for categorical data or the Kruskal-Wallis for the summative indices.

Appendix Table 3. Proportion Hospitalized (Yes) or Seroconverted (Yes) By Exposure Level Within Race/Ethnicity Strata*

Category	Overall (N = 3,422)	White NH (N = 2,312)	Hispanic (N = 500)	Black NH (N = 279)	API NH (N = 219)	Other NH (N = 112)	Chi-Square p value for Differences by Race/Ethnicity
Seroconversion - N(%)	161 (4.70)	93 (4.02)	37 (7.40)	17 (6.09)	7 (3.20)	7 (6.25)	<0.01
Potential exposure							<0.001
Less exposure risk	86 (3.73)	53 (3.26)	20 (6.43)	6 (4.03)	5 (3.33)	2 (2.78)	
More exposure risk	75 (6.73)	40 (5.82)	17 (8.99)	11 (8.46)	2 (2.90)	5 (12.50)	
Susceptibility							0.03
Less susceptible	130 (4.95)	76 (4.36)	30 (7.46)	13 (6.47)	7 (3.43)	4 (5.13)	
More susceptible	31 (3.90)	17 (2.98)	7 (7.14)	4 (5.13)	0	3 (8.82)	
Healthcare Access							0.02
Less barriers to access	93 (4.21)	58 (3.66)	21 (7.42)	8 (5.06)	3 (2.46)	3 (5.00)	
More barriers to access	68 (5.61)	35 (4.82)	16 (7.37)	9 (7.44)	4 (4.12)	4 (7.69)	
	Overall (N = 6,740)	White NH (N = 1,403)	Hispanic (N = 1,308)	Black NH (N = 899)	API NH (N = 465)	Other NH (N = 222)	Chi-Square P-Value for Differences by Race/Ethnicity
Hospitalization	401 (5.95)	185 (4.81)	108 (8.26)	78 (8.68)	13 (2.80)	17 (7.66)	<0.001
Potential exposure							<0.001
Less exposure risk	178 (4.30)	99 (3.85)	35 (4.95)	28 (6.41)	9 (3.01)	7 (5.51)	
More exposure risk	223 (8.59)	86 (6.76)	73 (12.15)	50 (10.82)	4 (2.41)	10 (10.53)	
Susceptibility							<0.001
Less susceptible	258 (4.88)	119 (4.07)	65 (6.07)	53 (7.60)	11 (2.53)	10 (6.13)	
More susceptible	143 (9.84)	66 (7.14)	43 (18.07)	25 (12.38)	2 (6.67)	7 (11.86)	
Healthcare access							<0.001
Less barriers to access	130 (3.52)	78 (3.23)	26 (4.65)	18 (4.63)	4 (1.71)	4 (4.35)	
More barriers to access	271 (8.89)	107 (7.48)	82 (10.95)	60 (11.76)	9 (3.90)	13 (10.00)	

*Chi-square assessed differences in proportion by White NH and Non-White.

Appendix Table 4. Modification of the association between race/ethnicity and seroconversion by potential SARS-CoV-2 exposure risk, susceptibility, and healthcare access – N = 3,086*

	White NH		Hispanic/Latino/a or Black NH		
Measure of Exposure	N Hospitalized /Denominator (%)	AOR (95% CI)	N Hospitalized /Denominator (%)	AOR (95% CI)	AOR (95% CI) for race/ethnicity effect within exposure strata; Hispanic/Latino/a or Black NH Versus White
Less exposure risk	53/1625 (3.26)	1.00	26/460 (5.65)	1.67 (1.02, 2.75)	1.67 (1.02, 2.75)
More exposure risk	40/687 (5.82)	1.64 (1.06, 2.54)	28/319 (8.78)	2.46 (1.47, 4.12)	1.50 (0.90, 2.52)
AORs (95% CI) for less versus more within strata of race/ethnicity		1.64 (1.06, 2.54) p = 0.02		1.47 (0.84, 2.60) p = 0.18	
RERI (95% CI): measure of interaction on the additive scale				0.15 (−1.16, 1.46) p = 0.82	
Susceptibility					
Less susceptible	76/1,742 (4.36)	1.00	43/603 (7.13)	1.59 (1.07, 2.37)	1.59 (1.07, 2.37)
More susceptible	17/570 (2.98)	0.72 (0.42, 1.25)	11/176 (6.25)	1.43 (0.74, 2.78)	1.98 (0.90, 4.33)
AORs (95% CI) for more versus less within strata of race/ethnicity		0.72 (0.42, 1.25) p = 0.24		0.90 (0.45, 1.78) p = 0.76	
RERI (95% CI): measure of interaction on the additive scale				0.11 (−0.97, 1.190) p = 0.84	
Healthcare					
Less barriers to access	58/1,586 (3.66)	1.00	29/441 (6.58)	1.67 (1.04, 2.68)	1.67 (1.04, 2.68)
More barriers to access	35/726 (4.82)	1.20 (0.77, 1.87)	25/338 (7.40)	1.80 (1.07, 3.03)	1.50 (0.88, 2.57)
AORs (95% CI) for less versus more within strata of race/ethnicity		1.20 (0.77, 1.87) p = 0.43		1.08 (0.61, 1.89) p = 0.55	
RERI (95% CI): measure of interaction on the additive scale				−0.07 (−1.15, 1.02) p = 0.89	

*aOR, adjusted odds ratio. Shown are Model adjusted for age, presence of children in the household, employment, income, race/ethnicity; Model adjusted for employment, income, race/ethnicity; and Model adjusted for age, employment, sex, income, race/ethnicity.

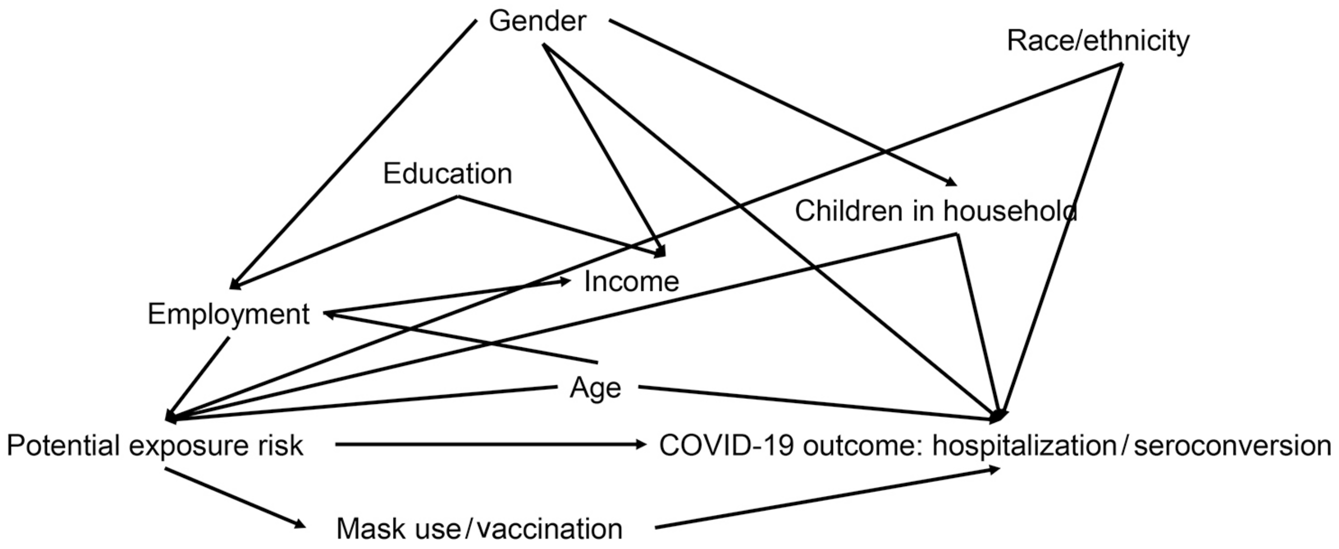

Appendix Figure 1. Directed acyclic graph depicting the relationship between potential SARS-CoV-2 exposure and COVID outcomes.

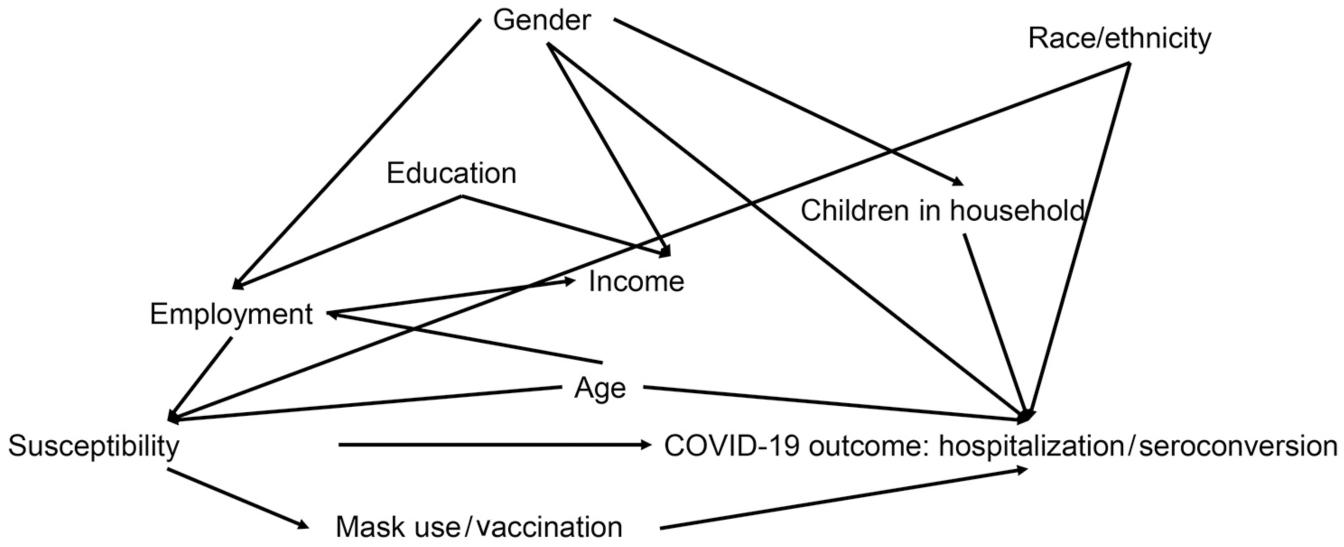

Appendix Figure 2. Directed acyclic graph depicting the relationship between susceptibility and COVID outcomes.

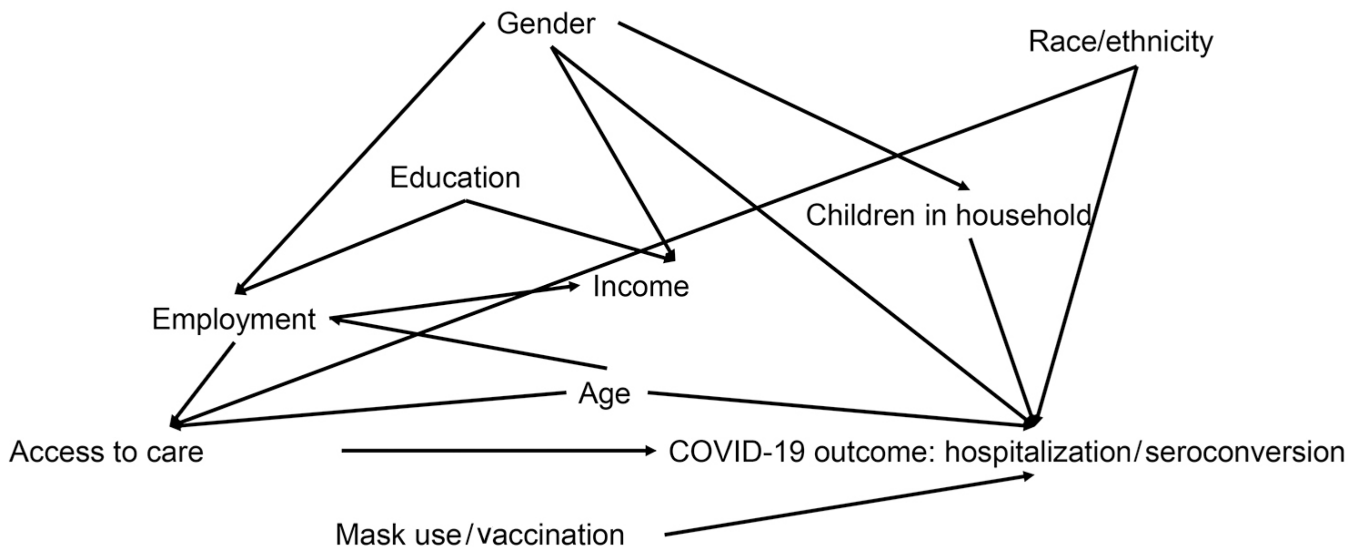

Appendix Figure 3. Directed acyclic graph depicting the relationship between access to care and COVID outcomes.
